# Incentives for pregnant mothers during antenatal care for better maternal and neonatal health outcomes in low and middle income countries: A Systematic Review and Meta-Analysis

**DOI:** 10.12688/f1000research.159261.1

**Published:** 2024-12-09

**Authors:** Ramesh Holla, Rosemol Johnson, Nisha A Khader, Mithun Rao, Bhaskaran Unnikrishnan, Anju Sinha, Darshan BB, Ravishankar N

**Affiliations:** 1 Kasturba Medical College Mangalore, Manipal Academy of Higher Education, Manipal, Karnataka, 576104, India; 2 Division of Reproductive, Maternal and Child Health, Indian Council of Medical Research, Ansari Nagar, New Delhi, India; 3 Department of Biostatistics, Vallabhbhai Patel Chest Institute, University of Delhi, New Delhi, India

**Keywords:** Pregnancy, incentive-based interventions, public health interventions, trials, maternal outcomes, neonatal outcomes

## Abstract

**Background:**

Universal access to maternal, newborn, and child healthcare is essential for achieving Sustainable Development Goal 3, but the effectiveness of various incentive-based programs for pregnant mothers in low- and middle-income countries (LMICs) remains uncertain. Objective of this systematic review was to determine if incentive-based interventions influenced maternal and neonatal health outcomes.

**Methods:**

We conducted a search in various databases from inception. All incentive-based interventional studies conducted on pregnant women in LMICs were included. Evidence from the included trials was synthesized using risk ratios (RRs) to compare the outcomes between groups receiving incentives and not receiving incentives. The meta-analysis was conducted using random-effects model. We assessed the quality of the included studies using the Cochrane Risk of Bias 2.0 tool and reviewed the collected data to determine its suitability for meta-analysis. This study is registered in PROSPERO (CRD42021247681).

**Results:**

Of the 3,897 records that were identified, 11 met eligibility criteria, all of which exhibited varying degrees of risk of bias, ranging from high to some concerns. Analysis of maternal outcomes across studies revealed no significant differences in the likelihood of delivering at a healthcare facility (RR 1.13, 95% CI: 0.86 to 1.47) and in frequency of prenatal care (RR 0.99, 95% CI: 0.88 to 1.12) between intervention and control groups. However, high levels of statistical heterogeneity were observed for both outcomes indicating variability among study results. Similarly, analysis of tetanus vaccine coverage showed no significant difference between groups (RR 1.00, 95% CI: 0.92 to 1.08), with moderate statistical heterogeneity observed.

**Conclusion:**

The maternal and neonatal outcomes examined in this review did not have any significant differences in intervention group when compared to the control group. The interventions to address maternal health concerns need to follow a multifactorial approach. There is a need for extensive primary research studies in the future.

## Introduction

The global data suggests significant disparity in Maternal Mortality Ratio (MMR) and Neonatal Mortality Rates (NMR) across various countries and regions. According to the United Nations Inter-agency Group for Child Mortality Estimation (UN IGME) - 2020, children born in South Africa or Asia are more likely to die compared to children born in developed economies. ^1^ According to the Million Death Study in 2015, neonatal mortality in India was high in rural areas. It was largely attributed to premature births and low birth weights. ^2^ In view of the current trends, it will be difficult for India to achieve the Neonatal mortality rate (NMR) targets for the Sustainable Development Goals (SDG) 2030 and National Health Policy 2025 ^3^. A gap analysis has indicated that it would take an additional 12.5 years for Liberia to meet the 2030 SDG target of reduction of MMR to less than 70 per 10,0000 live births. Likewise, a delay of 12.9 years will be seen with NMR. ^4^ Countries like Mauritania, and Algeria too are far away from reaching the targets of SDG goals ^5^ Another study conducted by Iván Mejía-Guevara et al., based on assessments of survey data from 31 countries in the Sub-Saharan African region has demonstrated that only 2 countries out of the 31 would meet the target of NMR by 2030. The model also forecasts that 13 countries would achieve the target between 2030 to 2050; while the remaining 13 countries are likely to succeed after 2050. ^6^

### What is already known?

A systematic review on psychosocial interventions among pregnant women for smoking cessation found that incentives influenced smoking reduction but the effect on outcome measures pertaining to low birth weight, preterm births, and mean birth weight was not clear owing to small sample sizes. ^7^

A previously conducted systematic review has shown only limited evidence that incentives may improve the frequency of prenatal care. This evidence is based on 5 trials and participants were majorly drawn only from low-income communities in Central America and North America. ^8^ A review of literature by Morgan et al., in 2013 showed that various types of incentives increased the number of antenatal care visits based on evidence from various studies conducted in LMICs. This study examined the allocation of incentives, considering both the demand side involving patients and the supply side involving healthcare providers, government agencies, and other stakeholders. ^9^

In a systematic review published in 2014, the evidence regarding five modes of demand-side financing was evaluated, focusing on their impact on out-of-pocket expenditure, utilization of health services, and cost-effectiveness. In addition, the study assessed maternal, perinatal, infant mortality, and morbidity among the poor and socially excluded pregnant women from LMICs. The study found that financing modes like cash transfers, conditional cash transfers and vouchers targeted for pregnant women increased the institutional deliveries and utilisation of antenatal and postnatal services. Evidence concerning maternal and neonatal mortality was sparse owing to the small sample size and shorter follow-up periods. ^10^ This systematic review has considered quantitative studies and qualitative studies. The study fails to clarify the design of intervention studies involved in the reviews and makes it difficult to relate the effect size to the various types of interventions.

The evidence generated in some of these reviews regarding the effectiveness of the provision of incentives for pregnant women focuses on a limited number of maternal and neonatal health outcomes like low birth weight, frequency of prenatal care, maternal and neonatal mortality. These reviews do not investigate any of the incentive-based behaviour change interventions that are also known to play a role in the prevention of pregnancy-related complications. This could include incentive-based tobacco and alcohol cessation, diet control intervention studies among pregnant women, etc.

### Rationale: Why is this important?

For an effective continuum of care, it is essential to strengthen the link between the home, the primary health care facility and the referral centres. ^4^ Studies from various countries have shown that there is a higher risk of poor pregnancy-related outcomes when the antenatal care received is inadequate. ^11– 13^ In accordance with the revised antenatal care guidelines 2016, the WHO recommends a minimum of eight contacts to reduce perinatal mortality and pregnancy complications. ^14^ There are various SBCE (Social Behavioural Community Engagement) interventions that facilitate the mobilisation of individuals, their family members, and communities for improving maternal and newborn health. ^15^ Demand-side financing is one of the SBCE interventions to promote the utilisation of health services for better health outcomes. ^16^ These include vouchers, Cash transfers, temporary payments, etc.

As per our knowledge, there are no comprehensive reviews focussing on the effectiveness of various incentive-based interventions that are targeted only for pregnant women during the antenatal period regarding broader outcome measures like the proportion of pregnancy, childbirth-related complications, perinatal deaths and proportion of preterm deliveries in resource-constrained settings that follows rigorous methodology of Systematic reviews. We aim to fill in the gap based on the work undertaken in previous reviews regarding the outcomes and the methodology. It is known that behavioural economics has potential in the formulation of effective health policies. Designing the incentives accordingly is one of the tools in the behavioural approach. ^17^ This review of trials is thus essential to inform the effectiveness of incentive-based programmes targeted for pregnant women in LMICs. It will help the policy makers to utilise the resources more effectively and to integrate the evidence-based public health initiatives into the health system.

Our research questions are as follows:

1. Does the provision of incentives to pregnant mothers during the antenatal care period achieve better maternal and neonatal health outcomes than the absence of such services for pregnant women?

1a. What are the effects of incentives (any type) on the uptake of antenatal care services/utilisation of antenatal health care services (frequency of antenatal care, proportion of institutional delivery)?

1b. What are the effects of incentives (any type) on maternal and neonatal morbidity (proportion of preterm deliveries, low birthweight (less than 2500 g), proportion of antenatal and postnatal complications, compliance to IFA tablets intake and Inj TT coverage, coverage and utilisation of maternal incentive based nutritional interventions, cessation of smoking, alcohol, tobacco, or any other unhealthy behavioural practises)?

1c. What are the effects of incentives (any type) on maternal and neonatal mortality (neonatal deaths, maternal deaths)?

### Objectives of our review

• To determine if any of the incentive-based interventions had an effect on maternal outcomes (proportion of antenatal and postnatal complications, proportion of institutional delivery, frequency of antenatal care, maternal deaths, compliance to IFA tablets intake and Inj TT coverage, coverage and utilisation of maternal incentive based nutritional interventions, cessation of smoking, alcohol, tobacco or any other unhealthy behavioural practises)
• To determine if any of the incentive-based interventions had an effect on neonatal health outcomes (proportion of preterm deliveries, low birthweight (less than 2500 g), perinatal and neonatal deaths)

## Method

We have published the protocol for this systematic review ^18^ and followed the guidelines of the Preferred Reporting Items for Systematic Reviews and Meta-Analyses (PRISMA). ^19^

### Identification, selection, and eligibility criteria

We used the following Population, intervention, comparator, and outcome (PICO) format to assess and select articles.

1. Population: We have included all incentive-based interventional studies conducted on pregnant women in LMICs. We have adhered to the definition of low-middle-income economies as per World Bank 2019, “lower-middle-income economies are those with a Gross National Income (GNI) per capita between $1,026 and $3,995”. ^20^ We excluded studies conducted among pregnant women who belong to low- and middle-income countries but reside in high-income countries during the antenatal and postnatal period. We have excluded studies that involve pregnant women living in developed countries but belonging to low-income families/communities. We define immigrants as people who migrate from one country to another country. So, women who hold citizenship in a low-middle-income country but reside in high, upper-middle and low-income countries during pregnancy will be excluded.
2. We excluded studies that focused on incentives targeted towards healthcare providers, government agencies or other supply stakeholders.
3. Intervention: We have included all interventions that consider incentives given to pregnant mothers linked to their antenatal care, which are usually not offered to pregnant mothers as standard prenatal care. We did not place any restrictions on the modes of financing such as conditional cash transfers, vouchers, transport services, in-kind goods, mama kits (basic supplies that are required at childbirth), co-payments etc.
4. Comparator: The interventions of our interest were compared to routine antenatal care (no incentives), no intervention or any other type of intervention that is not considered as incentive. We did not restrict the definition of usual care/routine antenatal care.
5. Outcomes: The outcomes of interest are categorised as neonatal outcomes and maternal outcomes. Neonatal outcomes of interest are as follows:
  • Proportion of preterm deliveries.
  • Low birthweight (less than 2500 g).
  • Perinatal and neonatal deaths.
Maternal outcomes are as follows:
  • Frequency of antenatal care (number of visits and the content of care)
  • Proportion of antenatal and postnatal complications.
  • Proportion of institutional delivery.
  • Maternal deaths.
  • Compliance to IFA tablets, intake and Inj TT coverage.
  • Coverage and utilisation of maternal incentive-based nutritional interventions.
  • Cessation of smoking, alcohol, tobacco, or any other unhealthy behavioural practices.

We have excluded studies that did not measure at least one of the above outcomes.

### Search strategy

The search strategy is added in the repository and is cited in the data availability statement. Searches were finalised and conducted on different databases from inception until 2021. We searched Medline, CINAHL, SCOPUS, Web of Science, and Embase for relevant records. The PICO acronym was used to describe the search strategy. Additionally, hand searching was carried out on the Cochrane Central database. Records were additionally retrieved from Google Scholar, and the reference lists of included studies were examined.

### Data collection and analyses

**Figure 1.**
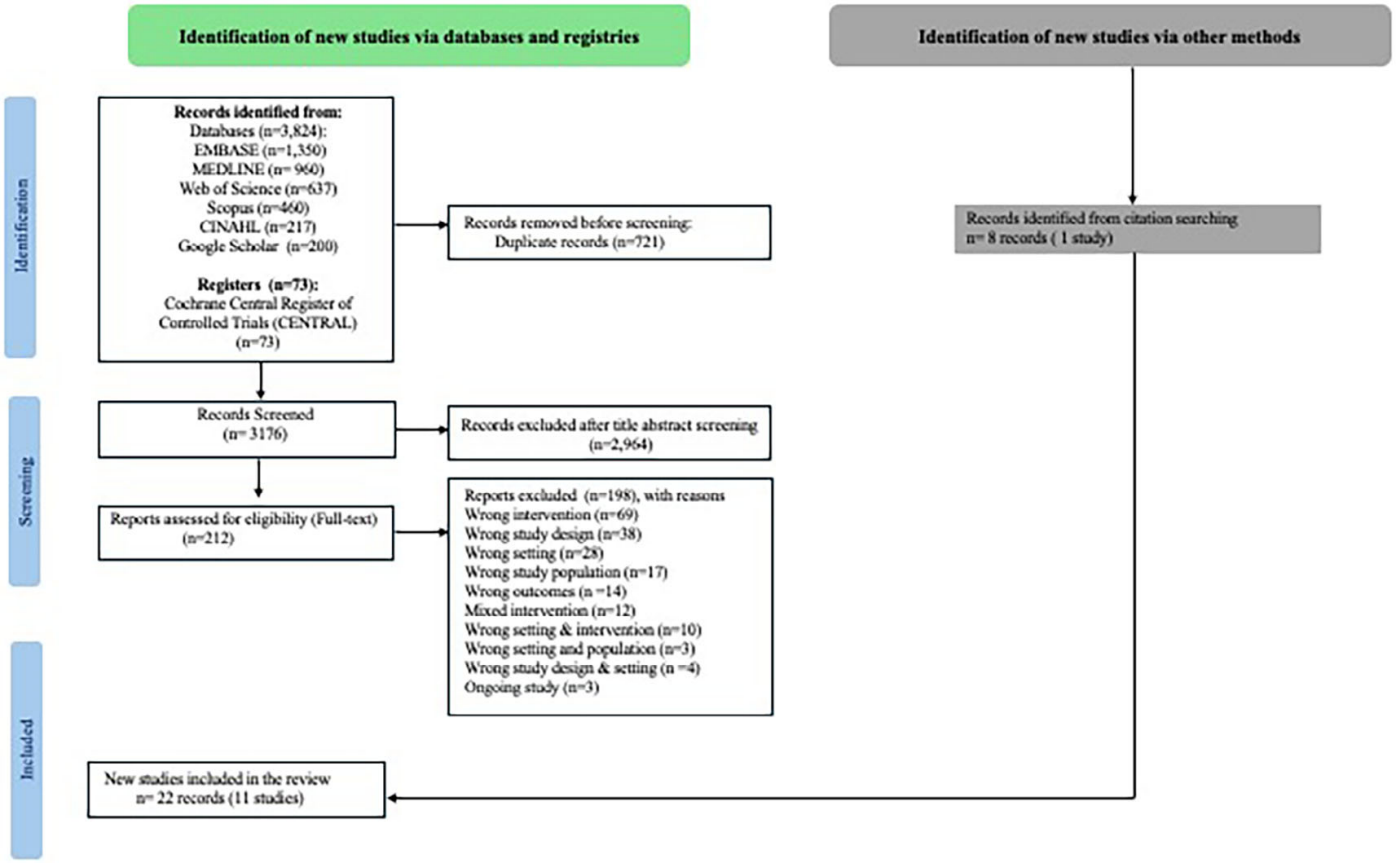
Identification of the included studies.

The screening process was conducted using the Covidence software ( https://www.covidence.org/). Initially, two reviewers screened titles, followed by abstract screening according to specified eligibility criteria. The full-text screening was then independently carried out by two reviewers. Ineligible records were excluded with specific reasons documented in flow chart ( Figure 1). Any discrepancies were resolved through discussions between the two reviewers. Additionally, we have documented ongoing trial protocols are added in the repository and is cited in the data availability statement. ^21– 23^

The data was extracted by one reviewer in the data extraction form under the following headings: study ID, author details, publication year, country details, participant details, intervention details, comparators, outcomes, type of incentive, funding details and type of setting (rural or urban). A second author verified the extracted information. The data was entered into Review Manager (RevMan) version 5 ( https://revman.cochrane.org/) (Cochrane Collaboration) by one author and verified by another author. Predefined outcomes that contained analysable data were as follows: Frequency of antenatal care, compliance to Inj TT coverage, the proportion of institutional delivery, proportion of women who received iron tablets and low birthweight.

The risk of bias was assessed using the tools mentioned in the Cochrane Handbook for Systematic Reviews of Interventions. ^24^ This assessment was independently conducted by two investigators. In case of disagreements, a third investigator was involved for the resolution of the same. The ROB-2 tool (version 2019) ( https://methods.cochrane.org/bias/resources/rob-2-revised-cochrane-risk-bias-tool-randomized-trials was used for the studies with randomised study designs. The variant of the ROB 2 tool adapted for) cluster randomised trials (2021 version) was used for cluster randomised trials. We evaluated and reported the results in ‘Risk of Bias’ tables. (The data on ‘Risk of Bias” tables has been added in the repository and be cited in the data availability statement)

We synthesized the evidence both narratively and graphically, using forest plots. Additionally, a meta-analysis was conducted for certain outcomes with available data. We calculated the risk ratio (RR) with a 95% confidence interval (CI) to compare outcomes between the two groups and performed a random-effects meta-analysis. Statistical heterogeneity was evaluated using the I ^2^ statistic, Tau ^2^, and the Chi ^2^ test.

## Results

### Characteristics of included studies

A total of 3824 records were imported into Covidence software ( https://www.covidence.org/) for screening, sourced from various databases: Embase (1350), CINAHL (217), Web of Science (637), Scopus (460), Medline (960), and Google Scholar (200). After removing 721 duplicates, 3103 records underwent screening based on their titles and abstracts using Covidence software. We could not export records from Cochrane (CENTRAL) to Covidence. Hence those records (73) were screened separately on Excel. We assessed 212 full texts for eligibility. We could retrieve 14 records (10 studies) on Covidence. In addition, 8 records (1 study) were found through backward and forward citation searches. Altogether we have included 11 studies for data extraction ( Figure 1). We did not find any studies that focussed on incentive-based behaviour change intervention conducted in LMICs during the antenatal care period among pregnant women for e.g. incentive-based smoking/tobacco cessation interventions, incentivised nutrition-focussed interventions for appropriate dietary behaviour during pregnancy, etc.

The detailed characteristics of the included studies are mentioned in the Table 1.

**Table 1.** Characteristics of included study.

Author, Year and Study Duration	Country	Study Design	Participant Characteristics	Intervention Details	Outcomes
**Barber, 2009** Study Duration: 6 Years	Mexico	Cluster-randomised controlled Trial	Women with a history of childbirth from 1997-2003 from low-income communities, either in the intervention or control group **Age**: 15-49 years	Cash transfers up to ~$15/month were provided. Additionally, households received bonuses for education. **Control group**: Received benefits after 2 years	Caesarean rate, delivery location, quality of prenatal care, birthweight, child growth, haemoglobin, cognitive development, language, and behavioural problems. **Tool used**: Administrative records from facilities and surveys
**Morris, 2004** Study duration: 24 months	Honduras	Cluster-randomised controlled trial	Pregnant women and mothers of children residing in areas recorded in a mid-2000 census **Age**: Not mentioned	Four groups: 1) Household-level package alone (received monthly vouchers worth 55 Lempiras) 2) Service-level package alone (quality improvement teams) 3) Both packages 4) Standard services (control) The service-level package was for system strengthening. Only household level data has been taken into account for this study	Adequate use of prenatal care (≥ 5 visits), postpartum check-up within 10 days of delivery, children <3 years taken to a health center in the past 30 days, immunisation rates, and growth monitoring. **Tool used**: Evaluation surveys (baseline and post-intervention)
**Okeke, 2019** Study duration: March 2017 to August 2018	Nigeria	Cluster-randomised controlled trial	Households with 1 ^st^ or 2 ^nd^ trimester pregnant women **Mean age:** Intervention: 24.8 years Control: 24.6 years	$14 payment was provided to households in the intervention group. Households in control group communities received gifts of nominal value at follow-up for participation	Overall child survival, foetal death, early infant death, neonatal deaths, and post-neonatal deaths **Tool used:** Baseline and follow-up interview
**Liu, 2019** Study duration: August 1, 2015 to April 19, 2017	Akwa Ibom, Nigeria	Individual-level randomised controlled trial	Pregnant women testing positive for HIV during antenatal care registration	HIV-positive women registering for ANC were eligible to receive up to 3 transfers during their pregnancy through 10 weeks after birth for achieving milestones: 7000 Naira (~US$24) after ANC registration plus 300 Naira (~US$1) of mobile phone credits (“Transfer 1”); 20,000 Naira (~US$70) when the participant gave birth at the same health facility where she registered for ANC (“Transfer 2”); and 6000 Naira (~US$20) when she returned to the facility to obtain an early infant diagnosis (EID) test for HIV (“Transfer 3”). Women in the control group received routine care.	Percentage of pregnant women who delivered their baby at the facility in which they were first enrolled for ANC, percentage of mothers who obtained an early infant diagnosis testing 6–8 weeks after giving birth to their child at the facility in which they were first enrolled for ANC. **Tool used:** Hospital records and additional surveys
**Briaux, 2020**	Northern Togo	Parallel cluster randomised controlled trial	Mothers and their children, 6-29 months old **Mean age**: Intervention: 29.3 years Control: 28.7 years	The intervention arm benefited from the ICCM-Nut program, CCPWs’ package of activities, and unconditional cash transfers (UCTs) (US$8.40/month) during their child’s “first 1,000 days” of life (from pregnancy to their second birthday) The control arm received benefits from the ICCM-Nut program along with the BCC activities only	HAZ, stunting (HAZ < −2 SD) among 6- to 29-month-old children, dietary diversity scores (DDSs) **Tool used:** Baseline and endline surveys
**Hemminki, 2021**	China	Cluster randomised trial	Pregnant women who utilised maternity care during the designated period were participants in the financial intervention arm **Age:** Not mentioned	Financial Intervention: Women were given 5 RMB if they had 1-3 check-ups and visit, RMB 10 for those who had 4-5 checkups and visits; RMB 15 for those with 6-7 checkups and visits, and RMB 20 for women with 8 checkups and visits Clinical intervention: Included three sessions of training in each county conducted by the obstetricians, gynaecologists and other health care professionals	First visit\3 months, prenatal visits 5, recommendation for hospitalisation, birth at higher level hospital, postnatal visits 1, content of care that includes breastfeeding 4+ (months), caesarean section, ultrasound 3+, anaemia test 1, blood pressure 3+, danger signs advice, no milk substitute **Tool used:** Mothers’ interviews were conducted to measure these outcomes.
**Handa, 2016**	Zambia	Cluster randomised trial	Mixed population of women and children below three years of age at program initiation [This program was specifically not designed to cater to the needs of pregnant women.]	A fixed amount of 60 Zambian kwacha was given on bimonthly basis to the primary female adult with children under the age of three	The program was primarily designed to evaluate the impact on child outcomes, including the following key areas: 1. Morbidity among children aged 0–60 months 2. Use of services among children aged 0–60 months 3. Nutritional status and feeding practices 4. Early childhood development indicators 5. Child needs being met 6. Education outcomes 7. Women’s decision-making For maternal utilization outcomes, the following were assessed: 1. Antenatal care (from a doctor or nurse) 2. At least four antenatal visits 3. Quality of antenatal care, defined by receiving Voluntary Counselling and Testing (VCT) for HIV, tetanus vaccination, and malaria treatment during antenatal care 4. Skilled attendance at birth (from a doctor or nurse) Tool used to measure outcomes: Baseline and follow-up surveys.
**Wang, 2016**	Zambia	Cluster randomised trial	Women who met the following criteria were included: 1. Delivered at a treatment facility during this time, or 2. Came to deliver at a treatment facility but were referred to another facility for medical reasons. **Mean age**: Intervention: 25.24 years Control: 24.79 years	Mama kit intervention consisted of items worth US$4 that include cloth (chitenge), baby diaper and blanket	Facility delivery **Tool used:** Semi structure interview for preference of items in mama kit intervention and administrative records from facilities
**Kahn, 2015**	Kisoro, Uganda	Multi-arm trial	Pregnant women over the age of 18 from selected villages were enrolled into the study when they presented for antenatal care at Muramba Health Centre Level III, KDH, or St. Francis Mutolere Hospital **Mean age**: Intervention groups: 25.4, 26.4, 23.8 years Control: 25.6 years	Modest cash transfers for participation in antenatal care. Intervention group 1: Cash incentives: 0.20 USD/ visit, Intervention group 2: Cash incentives: 0.40 USD/visit OR Intervention group 3: Cash incentives: 0.40 USD/once	Three or more antenatal visits and delivery in a health facility **Tool used:** Self-reports from participants and health care workers, record logs that were documented by midwives
**Grepin, 2019**	Kenya	Randomised controlled trial	1. Households with pregnant/lactating mothers or married women who were pregnant in the last two years 2. Households with children aged 6-15 years and 3. All other households [Note: we have included the study since the program targeted a mixed population that showed improved utilisation of health care among pregnant women]	PKH Program: Quarterly cash transfers ranging from Rp 200,000 to Rp 600,000 were given to households to address the issue of poverty PKH Consists of 1. Cash given to mothers quarterly; 2. Conditionality and cash penalty; 3. Field facilitators; and 4. Improvements in supply-side readiness. First, the cash, collected by mothers through the nearest post office, ranges from $60-220 per household per year depending on the number and age of children. The fixed amount is $20 per year. If a mother is pregnant and/or has children aged 0-6 years, she will receive additional $80 per year, regardless of the number of children. If a mother has one child at primary school, she will receive an additional $40 per year. If a mother has one child at secondary school, she will receive an additional $80 per year	Quality of human resources, poverty reduction, improved socio-economic condition of poor households, improved health and nutritional status of pregnant women, access to quality education and health **Tool used:** Surveys and qualitative studies
**Kusuma, 2016**	Indonesia	Cluster randomised controlled trial	Households (14,000 for PKH and 12,000 for Generasi) Women, with a focus on maternal health and higher-risk women **Age:** Not mentioned	The study did not have data pertaining to pregnant women alone. However, we have included the study since the program targeted a mixed population that showed improved utilisation of health care among pregnant women	Women's health knowledge, preferences for delivery locations, and financial barriers to accessing services, as well as the utilization of health services such as prenatal and postnatal visits, assisted deliveries, and facility deliveries

### Participant, study design and setting

Total number of pregnancies were 19565 from 10 studies. One study by Kusuma et al., ^25^ did not have data pertaining to pregnant women alone. However, we have included the study since the program targeted a mixed population that showed improved healthcare utilisation among pregnant women. The study duration ranged from 3 months to 6 years. Out of the 11 included trials, 8 studies were cluster RCTs ^25– 32^ and the remaining 3 studies were individually randomised parallel controlled trials. ^33– 35^

Participants in seven of the studies lived in rural areas ^26, 27, 30– 32, 34, 35^ while three had residents from rural, semi-rural or urban areas ^28, 29, 33^ One study had an urban population. ^25^ Four studies ^25, 27, 28, 31^ had a population comprising both pregnant women along with mothers and lactating women. These studies were included since they had done subgroup analysis for pregnant women participants. While seven studies had only pregnant women as participants. ^26, 29, 30, 32– 35^

All seven cluster RCTs have followed adequate accounting of cluster unit randomization in their analysis except Kusuma et al., 2016. ^25^ The studies included in the review were conducted in the following low- and middle-income countries: Uganda, Honduras, China, Kenya, Togo, Mexico, and Indonesia. Two of the studies were conducted in Zambia ^28, 32^ and two more in Nigeria. ^29, 33^ Eight studies were government-based interventions for the community. ^25, 27– 32, 34^ One of the studies was carried out by local Non-Governmental Organisations (NGOs) ^33^ while the other one was funded by an international agency. ^26^

### Maternal outcomes

We performed a meta-analysis for the three outcomes listed below, with the effects of the intervention shown in Table 2. Five RCTs did not have data for the outcome suitable for meta-analysis.

**Figure 2.**
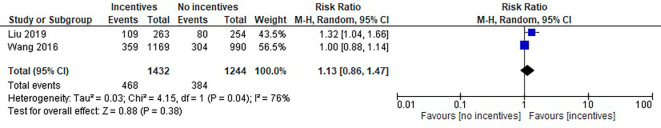
Effect of incentive-based intervention on the maternal outcome delivered at the facility.

**Figure 3.**
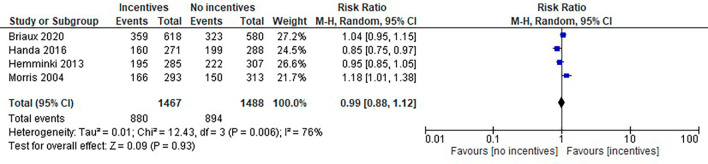
Effect of incentive-based intervention on frequency of prenatal care.

**Figure 4.**
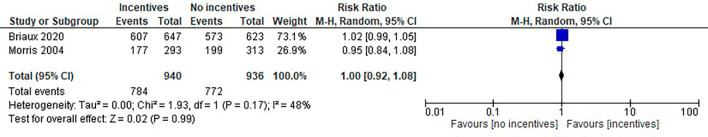
Effect of incentive-based intervention on tetanus vaccine.

1. **Delivered at the facility:** There was no clear difference between the two groups [Risk ratio (RR) 1.13, 95% confidence interval (CI) 0.86 to 1.47, 2676 women, two studies]. There was a high level of statistical heterogeneity. (I ^2^ = 76%, Tau ^2^ = 0.03 and Chi ^2^ test for heterogeneity P = 0.04) ( Figure 2).
2. **Frequency of prenatal care:** There was no clear difference between the two groups [RR 0.99, 95% CI 0.88 to 1.12, 2955 women, four studies]. There was a high level of statistical heterogeneity. (I ^2^ = 76%, Tau ^2^ = 0.01 and Chi ^2^ test for heterogeneity (P = 0.006) ( Figure 3). Another study provided details about prenatal visits, but we could not access the data due to its unavailability in the published reports. In the intervention households, the frequency of four or more prenatal visits increased by 4 points in percentage. Also, the likelihood of mothers completing 4 prenatal check-ups increased by 21 per cent. ^25^
  In another study by Kahn et al., the odds of women (odds ratio) attending three or more ANC visits was 1.7. ^34^
3. **Received Tetanus vaccine:** There was no clear difference between the two groups [RR 1.00, 95% CI 0.92 to 1.08, 1876 women, two studies]. There was a moderate level of statistical heterogeneity. (I ^2^ = 48%, Tau ^2^ = 0.00 and Chi ^2^ test for heterogeneity P = 0.17) ( Figure 4).

**Table 2.** Effect of interventions.

Outcomes of Interest	Effect size	Author (Year publication)
**Maternal Outcomes**		
**Delivered at the facility**	1.32 (1.04, 1.66)	Liu, 2021
	1.00 (0.88, 1.14)	Wang, 2016
**Frequency of prenatal care:**	1.04(0.95, 1.15)	Briaux, 2020
	0.85(0.75, 0.97)	Handa, 2016
	0.95(0.85, 1.05)	Hemminki, 2021
	1.18(1.01, 1.38)	Morris, 2004
**Received Tetanus vaccine:**	1.02[0.99, 1.05]	Briaux, 2020
	0.95 [0.84, 1.08]	Morris, 2004
**Compliance to IFA tablets intake:**	1.53 [0.98, 2.39]	Briaux, 2020
**Neonatal Outcome**		
**Low birthweight:**	1.58 [0.94, 2.64]	Briaux, 2020

Whereas, for the compliance to IFA tablet intake only one study has reported data for iron tablet intake by pregnant women, hence no meta-analysis could be performed. Also, no data was available for the outcomes such as maternal death, coverage and utilisation of maternal incentive-based nutritional interventions, proportion of antenatal and postnatal complications, and cessation of smoking, alcohol, tobacco, or any other unhealthy behavioural practices.

### Neonatal outcomes

1. **Low birthweight**: Only one study has given details about the same. Another study did not assess birthweight as its primary outcome. ^30^ The program showed a 127.3 g increase in birthweight among the intervention group and a reduction of 4.6 percentage in the incidence of low birth weight. ^30^
2. **Perinatal and neonatal deaths:** Only one study had given details about neonatal deaths. However, the data has not been adequate to conduct any analysis. The study has mentioned that the decrease in foetal deaths drove the increase in child survival. There was a reduction in foetal deaths, with a decrease ranging from 1.1 to 1.3 percentage points or a relative decrease of 23% compared to the control group. ^29^ The other study has not given specific details regarding mortality rates for newborns. Also, the definitions used in the study were not clear. The study showed that at baseline there were 0.17 mortality events (among 6- to 11-month-olds) per Kecamatan in control areas and only 0.12 mortality events in Program Keluarga Harapan (PKH) areas. At follow-up, those numbers had fallen to 0.09 and 0.08 respectively. So, there was no significant effect on the same. ^25^

## Risk of bias assessment

The risk of bias was independently assessed by two reviewers. We evaluated the risk of bias (ROB) of results from 6 RCTs that contributed to our analyses. We made no assessment of bias for 5 studies as they did not report relevant data or outcomes for this review. The completed RoB 2 tool with responses to all assessed signalling questions has been added in the repository and is cited in the data availability statement

1. **Frequency of prenatal care:** Among the four studies reporting this outcome, three were assessed to have some concern regarding risk of bias, while the remaining study was considered to have a high risk of bias. ^26– 28, 31^ In two of the studies, the protocol was unavailable, ^28, 31^ leaving uncertainty regarding potential deviations from the intended interventions, particularly in Handa’s study from 2016. ^28^ Morris’s study from 2004 ^31^ was judged to be at high risk of bias due to identified deviations in the study, attributed to lack of legal measures for transportation of resources and other challenges that they encountered during the study period. All of the studies were non-blinded since it is often not feasible in community-level interventions. Hence, they were all judged as having some concerns in risk of bias assessment.
2. **Received Tetanus Vaccine:** One of the studies had high risk of bias while another study had some concerns. ^27, 31^ Protocol was not available in one study. ^31^ While in the other study, there was no information on concealment of allocation sequence. ^27^ Hence the study was found to have some concerns of bias.
3. **Delivery in a health facility:** There was no information on the pre-specified analysis plans prior to the start of the study. Hence the study by Liu et al., 2019 was found to have some concerns of bias. ^33^ The study by Wang et al., was also found to have some concerns of bias because there was no adequate information regarding the randomization process. ^32^

Overall, these studies exhibited various concerns regarding risk of bias. Incentives differed, including vouchers, cash transfers, and mama kits, which may limit their generalizability across different communities in LMICs. Additionally, the insufficient data prevented us from conducting a subgroup analysis based on incentive types. The meta-analysis included fewer than ten studies, so we did not perform a funnel plot. There is a potential for publication bias, especially since we did not search trial registries in LMIC regions.

## Discussion

We retrieved 11 studies that involved incentives for pregnant women. Six studies examined the outcome frequency of prenatal care. However, only 4 of the studies had data that could be incorporated into meta-analysis. ^26– 28, 31^ The remaining 2 studies did not have data that could be analysed. ^25, 34^

Seven studies examined the outcome of delivered at the facility/skilled attendance at birth but only 2 studies could be incorporated into meta-analysis ^32, 33^ and 3 studies examined the receipt of tetanus vaccine during the antenatal period. However, only 2 of the studies could be incorporated into meta-analysis. ^27, 31^ In the study by Handa et al.,2016, ^28^tetanus vaccine is assessed as part of the quality of antenatal care assessment. There is no separate data on the same. There is no adequate evidence to show the impact of incentives on maternal and neonatal outcomes. Besides, data was available only for 3 of the outcomes of our interest. Only 6 studies had data that were suitable for meta-analysis. All the studies are from low- and middle-income countries. A study previously conducted in 2015, had included studies worldwide and had included only 5 studies. Only 2 studies out of the 5 had been conducted in low- and middle-income communities. ^14^ Our study, which was limited to low and middle-income countries, has included 11 studies in the review. Low birth weight was assessed only in one study by Briaux et al. ^27^ But it was assessed as the intermediary outcome in the study.

A systematic review conducted by Toolan et al. in 2021 investigated the impact of incentives, alongside other interventions, on maternal and neonatal health outcomes during the antenatal period. ^36^ The authors did not perform a meta-analysis due to the heterogeneity of interventions and outcomes, and instead presented their findings narratively.

Only two studies in this review examined the effect of cash incentives given to women who attended four or more antenatal care (ANC) visits. ^37, 38^ One of these studies was cross-sectional in nature, ^37^ while the other was qualitative in nature. ^38^ Bhatt et al.’s study found that both the maternal incentive scheme (MIS) and Aama policies significantly increased the utilization of four ANC visits and institutional deliveries in Nepal. ^37^ Flueckiger et al.’s study revealed that stakeholders, particularly mothers, perceived the monetary incentive as a motivating factor for attending ANC sessions, which led to increased attendance. In contrast, our study included six studies ^25– 28, 31, 34^ that assessed the frequency of prenatal care, but only four studies were included in the meta-analysis. While our study did not find a significant difference in the effect of intervention between the two groups, it is important to consider other factors that may have influenced the results, such as the duration of the intervention or the specific intervention strategies used in each group and the design of the study. While the study did not find a significant difference in the effect of intervention between the two groups, it is important to consider other factors that may have influenced the results, such as the duration of the intervention or the specific intervention strategies used in each group.

### Strengths and limitations of this study

The current systematic review has followed the methodology adopted in the Cochrane Handbook for Systematic Reviews of Interventions. ^39^ The results generated out of this systematic review will inform or guide in implementing public health interventions and policies about incentive-based initiatives for pregnant women.

No restrictions concerning the publication year were followed for the search strategy.

We have included all the financing modes implemented for pregnant women. There were no restrictions on the above. The modes of financing included vouchers, transport incentives, in-kind goods, cash transfers, etc.

The inclusion of studies was restricted to English and those published in scientific journals.

### Implications for research

The current review was limited to studies that were primarily concerned about demand-side incentives. In the future, studies should involve a combination of both demand and supply-side incentives. In addition, focus of trials should be on outcomes like neonatal and maternal mortality, postpartum complications, and skilled attendance at homes alongside institutional delivery.

It is likely that the very low number of studies in LMICs could be attributed to the very low incentives, investments, and infrastructure for research activities in the regions compared to high-income countries that leads to substantial disparities in the conduct of research studies. Studies on tobacco and smoking cessation were excluded since they were all conducted in high-income countries. ^40– 44^ Most of the studies in the current review were funded by various external sources and some were conducted by the government. While a few were done in collaboration with the national government with the support of external funding. To promote more studies in LMICs in the future, national governments should prioritize extensive primary research. ^45^ Communities need to be empowered to conduct community-based research activities that enable them to identify issues and develop solutions for the same. ^46^

The outcomes examined in this review did not have any significant differences in the intervention group when compared to the control group.

Evidence is limited to low-middle income economies only. The definitions of low-middle-income economies have changed substantially in the past two years and during the pandemic. ^47, 48^

We did not have enough resources to include studies conducted in languages other than English in LMICs. We could have missed out on the effects of studies conducted in LMICs in regional languages. Additionally, we did not search registries or explore grey literature sources.

The interventions to address maternal health concerns need to follow a multifactorial approach. Poverty is not the sole barrier for pregnant women to access healthcare services. Reports of obstetric violence/labour room violence have been commonly seen across India and the globe. ^49– 51^ Efforts to address the same needs to be taken. In addition, one needs to address the challenges faced by health care workers. ^52^ Besides these, other environmental and social factors also play a role in the health of pregnant women. Measures to address the same needs to be implemented.

## Conclusion

Despite the extensive search yielding only 11 eligible studies from low- and middle-income countries, the analysis revealed varying degrees of bias across the selected studies. Our examination of maternal outcomes, including facility delivery, prenatal care frequency, and tetanus vaccine coverage, demonstrated no significant differences between intervention and control groups. However, high levels of statistical heterogeneity were observed, indicating considerable variability among study results. These findings underscore the complexity of addressing maternal health concerns and suggest that interventions require a multifactorial approach. Moreover, the moderate statistical heterogeneity observed for tetanus vaccine coverage suggests the need for further exploration. One study reported a modest increase in birthweight and a reduction in low birthweight incidence, but outcomes related to perinatal and neonatal deaths were inconclusive. Prospectively, there is a critical need for comprehensive primary research studies to better inform strategies aimed at improving maternal and neonatal health outcomes.

## Reporting guidelines

**Figshare:** PRISMA checklist and flowchart: Incentives for pregnant mothers during antenatal care for better maternal and neonatal health outcomes in low and middle income countries: A Systematic Review and Meta-Analysis, https://doi.org/10.6084/m9.figshare.27916326.v1; https://doi.org/10.6084/m9.figshare.27915747.v2.

Data are available under the terms of the Creative Commons Attribution 4.0 International license (CC-BY 4.0).

## Ethics and consent

Ethical approval and consent were not required.

## Data Availability

No data are associated with this article. Figshare repository- “Extended data of “Incentives for pregnant mothers during antenatal care for better maternal and neonatal health outcomes in low and middle income countries: A Systematic Review and Meta-Analysis””. This project contains the following extended datasets ^53– 55^:
• Extended data of SR Incentive. (Contains search strategy, ROB tool and the ongoing protocol details.) 10.6084/m9.figshare.27916326. • PRISMA Flowchart of SR incentive. (Contains PRISMA flowchart of the systematic review.) 10.6084/m9.figshare.27915747. • PRISMA checklist of SR Incentive. (Contains PRISMA checklist of the systematic review) 10.6084/m9.figshare.27916326. Data are available under the terms of the Creative Commons Attribution 4.0 International license (CC-BY 4.0).
