# Supplementary material for: Incentives for pregnant mothers during antenatal care for better maternal and neonatal health outcomes in low and middle income countries: A Systematic Review and Meta-Analysis
Source: F1000Res. 2025 May 29;13:1512. Originally published 2024 Dec 9. [Version 2] doi: 10.12688/f1000research.159261.2 (PMC12511867; doi:10.12688/f1000research.159261.2)
Supplement: Peer review history

# Reviewer report: Reviewer response for version 2

**DOI:** 10.5256/f1000research.179546.r418444

**Published:** 2025-10-09

**Authors:** Rohith Motappa

**Affiliations:** 1 ESIC Medical College & PGIMSR and Model Hospital, Bangalore, Karnataka, India

Introduction: The author can explain a little more on the SDG goals related to the article a little more making the topic highly relevant. Not only this makes the article relevant but also helps in catching the attention of the readers. Few places abbreviations are present in the document without proper expansion. Please make sure that all the abbreviated forms are expanded when it is being used for the first time in the document. The end of the introduction section has mentioned about few less known countries. Either the article can mention about the continent where it is falling in or socio-economic strata they fall into (Underdeveloped/developing). Objectives again contain the abbreviations related to the common medications that are being given. Please expand it so that it is readable as they are objectives.

 Methodology: Whether all the articles have been included for the study or the only ones in English language has been considered for the study. Please explicitly mention the same. Search strategy abbreviations have to be expanded.

 Results: The section can undergo a thorough grammar check and modify the sentences where singular has been used in place of plural and vice versa. The reason for exclusion of the studies have to be clearly mentioned in the result section of the document. The subheadings under the maternal outcomes have quoted the relevant statistics extracted from the individual studies but due referencing is not given. Please make sure the citations are done for the individual studies in the result section. Grammar check need to be done In risk of bias, where the sentences can be paraphrased, for ex: We made no assessment of bias for 5 studies as they did not report relevant data or outcomes for this review, can be made as Assessment of risk of bias was not conducted for 5 studies as the studies did not report relevant outcomes.

 Discussion has been well written where this systematic review has been compared with the other studies of the past and also underlined the importance of the Systematic review. Wherever the results are repeatedly being told, the statistics can be minimized. If the author

 feel that there is necessity to highlight the statistics again, then can be kept. The limitation that only published articles were considered need to be mentioned as one of the limitations of the study. Please check the reference list for the referencing style.

Are the rationale for, and objectives of, the Systematic Review clearly stated?

Yes

Is the statistical analysis and its interpretation appropriate?

Yes

If this is a Living Systematic Review, is the ‘living’ method appropriate and is the search schedule clearly defined and justified? (‘Living Systematic Review’ or a variation of this term should be included in the title.)

Not applicable

Are sufficient details of the methods and analysis provided to allow replication by others?

Partly

Are the conclusions drawn adequately supported by the results presented in the review?

Partly

Reviewer Expertise:

NCDs, TB and Geriatrics.

I confirm that I have read this submission and believe that I have an appropriate level of expertise to confirm that it is of an acceptable scientific standard.

# Reviewer report: Reviewer response for version 2

**DOI:** 10.5256/f1000research.179546.r418442

**Published:** 2025-10-07

**Authors:** Mohammed Shoyaib Khazi

**Affiliations:** 1 All India Institute of Medical Sciences, Mangalagiri, Andhra Pradesh, India

While PRISMA is mentioned, several required elements are missing. The **PRISMA flow diagram** is inadequately labeled (Figure 1 should clearly show the number excluded at each stage and reasons). Please add total number of studies at each stage. 

 The **meta-analysis includes only 2–4 studies per outcome**, which undermines the robustness of pooled estimates; kindly provide the reason for the same or mention this as limitation.

 The conclusion that “incentives showed no significant benefit” should be contextualized, could this be due to short intervention duration, low incentive value, or weak health system readiness?

 The manuscript contains several grammatical errors and inconsistencies in referencing style.

 The paragraph content can clearly mention the important results.

**Minor Comments**

 1. Abbreviations (e.g., ANC, IFA, TT) should be defined at first mention in both abstract and text.

 2. Reference numbering and formatting need standardization per journal guidelines.

 3. Figures should have complete captions including data sources.

 4. Some references (e.g., SDG targets) are outdated; update to the latest WHO/UN data.

 5. The results for neonatal outcomes such as low birth weight and perinatal mortality are mentioned in objectives but not presented, clarify if data were unavailable or excluded.

Are the rationale for, and objectives of, the Systematic Review clearly stated?

Yes

Is the statistical analysis and its interpretation appropriate?

Yes

If this is a Living Systematic Review, is the ‘living’ method appropriate and is the search schedule clearly defined and justified? (‘Living Systematic Review’ or a variation of this term should be included in the title.)

Yes

Are sufficient details of the methods and analysis provided to allow replication by others?

Yes

Are the conclusions drawn adequately supported by the results presented in the review?

Yes

Reviewer Expertise:

Community Medicine, Public Health, Demographic Health Surveys

I confirm that I have read this submission and believe that I have an appropriate level of expertise to confirm that it is of an acceptable scientific standard.

# Reviewer report: Reviewer response for version 1

**DOI:** 10.5256/f1000research.174960.r361421

**Published:** 2025-02-24

**Authors:** Nicola McMeekin

**Affiliations:** 1 Glasgow Institute of Health & Wellbeing, University of Glasgow, Glasgow, Scotland, UK

Thank you for the opportunity to review this interesting paper. It reports the findings of a systematic review that searched for papers evaluating the impact on maternal and neonatal outcomes of offering  incentives to pregnant women in low and middle income countries.  

 Overall this paper is very well written and interesting to read. My suggestions for edits are mainly for overall clarification not specific issues, to set the scene for the reader before you go into your well written sections. This is often the case when you know you subject well, but a few small clarifications and scene setting sentences would improve what is interesting and relevant research.

**Minor**

**Introduction**

 Please could you move the detail of the SDG (MMR less than 70 per 100000 live deaths) to earlier on in the first paragraph in the Introduction? At the moment the reader has to wait till the sentence about Liberia to find out what the detail is.

 I think this section would benefit too from a sentence or two on the overall issue with LMICs – you start off with describing South Africa and Asia in the second sentence. Could you add a bit to the first sentence along the lines of ‘…..countries and regions, particularly between higher and LMICs’ – or something similar.

 I was also confused by the last sentence in the first paragraph: ‘ *The model* *also forecasts that 13 countries would achieve the target between 2030 to 2050; while the remaining 13 countries are likely to succeed after 2050’. *In the previous sentence you stated that the study looks at data from 31 countries, the 2 comments about 13 countries would suggest that the study you cite looks at 26 countries.  Please could you clarify why the 2 amounts in your last sentence don’t add up to 31?

 In the Introduction you focus on maternal and neonatal mortality rates. It is not immediately obvious to me why you have chosen these outcomes to discuss. Please consider adding a short explanation of why you chose these specific outcomes, you might say that they are key outcomes that are linked to other important maternal/neonatal outcomes, or that they are proxy’s for other outcomes? For example in the second paragraph of the ‘Rationale: Why is this important?’ section in the Introduction in the first sentence you introduce some relevant outcomes such as pregnancy and childbirth-related complications, maybe introduce some of these in the first paragraph of the Introduction?

 The ‘What is already known’ section could benefit from a short introduction paragraph. At the moment it starts with smoking cessation incentives, it would be good to set the scene with saying something along the lines of how incentives could benefit this area (in terms of reducing smoking and improving engagement with antenatal care for example) and improve maternal and child outcomes. This would help orientate the reader to what to expect in this section before it starts with more specific details. The last paragraph closes this section really nicely, starting with something similar as suggested would benefit it.  

 In the research questions section please could you write out in full what ‘IFA’ and ‘Inj TT’ refer to in 1b?

**Methods**

 The Methods section is clear and very well written, it included everything I would expect to see in a systematic review. I was unable to see the search strategy in the repository – only PRISMA checklists and flowchart. Please could you check that the strategy is included – I just many not be able to see it - this is why I answered 'partly' to the question - Are sufficient details of the methods and analysis provided to allow replication by others?

 In Outcomes – please could you clarify your definition of preterm delivery – i.e. born alive before 37 weeks, also for perinatal and neonatal deaths, and maternal deaths.

**Discussion**

 Fourth paragraph in the Discussion section – the last 2 sentences are very similar – could this be a duplication?

 Would it be possible to highlight that previously there were a small number of studies and with small sample sizes – and this study has not found a large number of studies  – but most studies were RCTs – so assumably suitably powered? It would be good to have that additional information.

 I really liked your last paragraph in the Discussion section about poverty not being the sole barrier for pregnant women to access healthcare services. Would it be possible to add another sentence or two to this paragraph if there is more evidence available similar to the violence you discussed in the second sentence? 

 In the  ‘Implications for research’ section please could you consider moving the 4 ^th^ and 5 ^th^ paragraphs (evidence limited to LMIC and limited study resources for searching for non-English records) to the ‘Strengths and limitations of the study’ section – they read more like strengths and limitations rather than implications.

**Typos/grammar**

 I only found a few typos/grammatical errors and mainly in the Results section:

 In ‘Neonatal outcomes – 2. Perinat and neonatal deaths’ – ‘the data has not been adequate to conduct any analysis’ would read better as ‘the data was not adequate to conduct any analysis’

 You also mix present and past tense in the Discussion:

 Second paragraph – ‘there is no separate data on the same’ should read ‘there was no separate data on the same’, and ‘there is no adequate evidence to show the impact of incentives…’ should read ‘there was no adequate evidence to show the impact of incentives……’

Are the rationale for, and objectives of, the Systematic Review clearly stated?

Yes

Is the statistical analysis and its interpretation appropriate?

Yes

If this is a Living Systematic Review, is the ‘living’ method appropriate and is the search schedule clearly defined and justified? (‘Living Systematic Review’ or a variation of this term should be included in the title.)

Not applicable

Are sufficient details of the methods and analysis provided to allow replication by others?

Partly

Are the conclusions drawn adequately supported by the results presented in the review?

Yes

Reviewer Expertise:

Health economics

I confirm that I have read this submission and believe that I have an appropriate level of expertise to confirm that it is of an acceptable scientific standard, however I have significant reservations, as outlined above.

# Response

**Published:** 2025-03-17

**Authors:** Ramesh Holla

**Affiliations:** Kasturba Medical College, Mangalore, Manipal Academy of Higher education, Manipal, India, India

Point to point response to the reviewer’s comments:      

 1.    Comment: Please could you move the detail of the SDG (MMR less than 70 per 100,000 live births) to earlier on in the first paragraph in the Introduction? At the moment, the reader has to wait till the sentence about Liberia to find out what the detail is.

 Author’s response: Thank you for your suggestion. We have moved the SDG detail (MMR less than 70 per 100,000 live births) to the first paragraph of the Introduction for better clarity and immediate context. 

 We rephrased the original paragraph to "The Million Death Study (2015) found that neonatal mortality in India was particularly high in rural areas, largely attributed to premature births and low birth weights (6). In view of the current trends, it will be difficult for India to achieve the NMR targets for SDG-2030 and National Health Policy 2025 (7). A gap analysis indicated that Liberia would take an additional 12.5 years beyond 2030 to meet the SDG target for MMR, with a similar 12.9-year delay projected for NMR (8)."to maintain readability.

 Action taken in the manuscript: The change can be seen in the “Introduction” section, where the SDG goal is now introduced earlier in the first paragraph of the Introduction. 

 The rephrased sentences can be found in the last two paragraphs of introduction section

 2.    Comment: I think this section would benefit too from a sentence or two on the overall issue with LMICs – you start off with describing South Africa and Asia in the second sentence. Could you add a bit to the first sentence along the lines of ‘.....countries and regions, particularly between higher and LMICs’ – or something similar.

 Author’s response: Note your suggestion. We have added a phrase to the first sentence of the Introduction to emphasize disparities between higher-income countries (HICs) and low-and-middle-income countries (LMICs).

 Action taken in the manuscript: The change can be seen in the “Introduction” section, where the first sentence of the introduction section now highlights disparities between HICs and LMICs. 

 3.    Comment: I was also confused by the last sentence in the first paragraph: ‘The model also forecasts that 13 countries would achieve the target between 2030 to 2050; while the remaining 13 countries are likely to succeed after 2050’. In the previous sentence you stated that the study looks at data from 31 countries, the 2 comments about 13 countries would suggest that the study you cite looks at 26 countries. Please could you clarify why the 2 amounts in your last sentence don’t add up to 31?

 Author’s response: We have added a clarification that mortality projections were unavailable for three countries due to data quality limitations to ensure the total sums up to 31.

 Action taken in the manuscript: The change can be seen in introduction section, last line of the fourth paragraph

 4.    Comment: In the Introduction you focus on maternal and neonatal mortality rates. It is not immediately obvious to me why you have chosen these outcomes to discuss. Please consider adding a short explanation of why you chose these specific outcomes, you might say that they are key outcomes that are linked to other important maternal/neonatal outcomes, or that they are proxies for other outcomes

 Author’s response: Thank you for your valuable comment. We have added an explanation stating that maternal and neonatal mortality rates are key indicators of healthcare system effectiveness, reflecting both direct health outcomes and broader factors such as healthcare accessibility, service quality, and social health determinants. These statistics also serve as indicators of pregnancy-related complications, perinatal health risks, and the efficacy of healthcare interventions

 Action taken in the manuscript: The change can be seen in second paragraph of the Introduction, where this explanation has been added. New references 2 and 3 has been added.

 5.    Comment: For example, in the second paragraph of the ‘Rationale: Why is this important?’ section in the Introduction, in the first sentence, you introduce some relevant outcomes such as pregnancy and childbirth-related complications. Maybe introduce some of these in the first paragraph of the Introduction?.

 Author’s response: Added a sentence introducing pregnancy-related complications, including miscarriage, preterm labour, stillbirth, and maternal or neonatal morbidity and mortality, emphasizing that these complications can occur at any stage of pregnancy, during labour, or postpartum

 Action taken in the manuscript: The change can be seen in the “Introduction” section, and a new reference (4) has been added

 6.    Comment: The ‘What is already known’ section could benefit from a short introduction paragraph. At the moment, it starts with smoking cessation incentives. It would be good to set the scene with something along the lines of how incentives could benefit this area (in terms of reducing smoking and improving engagement with antenatal care, for example) and improve maternal and child outcomes. This would help orient the reader to what to expect in this section before it starts with more specific details.

 Author’s response: Added an introductory paragraph explaining that incentive-based interventions, including conditional cash transfers (CCTs) and vouchers, have been implemented globally with some success in maternal and neonatal health programs. These initiatives aim to overcome healthcare access barriers, improve maternal health practices, and enhance birth outcomes

 .

 Action taken in the manuscript: The change can be seen in the first paragraph under the "What is already known" section, and new references (11–21) have been added 

 7.    Comment: In the research questions section please could you write out in full what ‘IFA’ and ‘Inj TT’ refer to in 1b.

 Author’s response: Expanded the abbreviations to Iron and Folic Acid (IFA) tablets intake and Tetanus Toxoid injection (Inj TT) for clarity

 Action taken in the manuscript: The change can be seen in 1b in “Our Research Questions” Section

 8.    Comment: I was unable to see the search strategy in the repository – only PRISMA checklists and flowchart.

 Author’s response: The search strategy was shared via Figshare and is available under the Data Availability section 

 Action taken in the manuscript: No changes were made

 9.    Comment: In Outcomes – please could you clarify your definition of preterm delivery – i.e. born alive before 37 weeks, also for perinatal and neonatal deaths, and maternal deaths

 Author’s response: Thank you for the suggestion. We have incorporated the clear definitions for preterm delivery, low birthweight, perinatal deaths, and neonatal deaths

 Action taken in the manuscript: The change can be seen in first few lines under the Outcomes section, with new references (36–39) added

 10.    Comment: Fourth paragraph in the Discussion section – the last 2 sentences are very similar – could this be a duplication.

 Author’s response: Identified and removed duplicate sentences in the fourth paragraph of the Discussion section

 Action taken in the manuscript: The change can be seen in the “Discussion” section, where the duplicated content has been deleted

 11.    Comment: Would it be possible to highlight that previously there were a small number of studies and with small sample sizes – and this study has not found a large number of studies – but most studies were RCTs – so assumably suitably powered? It would be good to have that additional information.

 Author’s response: Added a clarification that previous reviews had a limited number of studies with small sample sizes, reducing their reliability. Our review also did not find a significantly larger number of studies; however, most were RCTs, which are generally well-powered to detect meaningful effects, strengthening the evidence base

 Action taken in the manuscript: The change can be seen in last paragraph of the “Discussion” section

 12.    Comment: Would it be possible to add another sentence or two to this paragraph if there is more evidence available similar to the violence you discussed in the second sentence?

 Author’s response: Expanded the paragraph to include additional barriers to maternal healthcare access, such as challenges faced by healthcare workers, patriarchal norms limiting women’s autonomy, cultural preferences for home births, and poor healthcare infrastructure

 .

 Action taken in the manuscript: The change can be seen in last paragraph under the "Implication of Research" section, with new references (71–79) added

 13.    Comment: In the ‘Implications for research’ section, please could you consider moving the 4th and 5th paragraphs (evidence limited to LMICs and limited study resources for searching for non-English records) to the ‘Strengths and limitations of the study’ section – they read more like strengths and limitations rather than implications.

 Author’s response: Thank you for your valuable comment. We have moved the 4th and 5th paragraphs from the "Implications for Research" section to the "Strengths and Limitations of the Study" section for better alignment

 .

 Action taken in the manuscript: The change can be seen in the "Strengths and Limitations" section

 14.    Comment: In ‘Neonatal outcomes – 2. Perinatal and neonatal deaths’ – ‘the data has not been adequate to conduct any analysis’ would read better as ‘the data was not adequate to conduct any analysis’. You also mix present and past tense in the Discussion: Second paragraph – ‘there is no separate data on the same’ should read ‘there was no separate data on the same’, and ‘there is no adequate evidence to show the impact of incentives...’ should read ‘there was no adequate evidence to show the impact of incentives......’.

 Author’s response: Revised the text to ensure consistent past tense usage: "No separate data was available, and there was no adequate evidence to show the impact of incentives on maternal and neonatal outcomes.".

 Action taken in the manuscript: The change can be seen in second paragraph of the “Discussion” section.
